# The association between *TNP2* gene polymorphisms and Iranian infertile men with varicocele: A case-control study

**DOI:** 10.18502/ijrm.v17i8.4821

**Published:** 2019-09-03

**Authors:** Mohammad Mehdi Heidari, Amirhossein Danafar, Fahime Moezzi, Mehri Khatami, Ali Reza Talebi

**Affiliations:** ^1^Department of Biology, Faculty of Science, Yazd University Yazd Iran.; ^2^Department of Biology, Ashkezar Islamic Azad University AshkezarYazd Iran.; ^3^Research and Clinical Center for Infertility and Department of Anatomy, Shahid Sadoughi University of Medical Sciences Yazd Iran.

**Keywords:** Varicocele, Genetic polymorphism, Infertility, Nuclear transition protein 2

## Abstract

**Background:**

Numerous researches have provided great evidence that revealed the relationship between varicocele and sperm DNA damage.

**Objective:**

Because of the crucial role of nuclear transition proteins (TPs) in sperm DNA condensation and integrity, this case-control study was designed to study *TNP2* gene nucleotide variations in Iranian patients with varicocele.

**Materials and Methods:**

PCR-SSCP and DNA sequencing were used to search for mutations in exons 1 & 2 of the *TNP2* gene in 156 infertile patients with varicocele and 150 fertile men.

**Results:**

The results of sequencing showed three variants at positions c.301C░>░T (p.R101C), c.391C░>░T (p.R131░W), and g.IVS1-26G░>░C (rs8043625) of *TNP2* gene. It was found that varicocele risk in men who have the CC genotype of g.IVS1-26G░>░C SNP is higher than those who don't have these genotypes (according to Co-dominant model, Dominant model, Recessive model, and Over-dominant model). The haplotype-based analysis showed that (C/C/T) and (C/T/T) haplotypes were a risk factor of in patients with varicocele compared to controls (OR░=░3.278, p░=░0.000 and OR░=░9.304, p░=░0.038, respectively).

**Conclusion:**

Because of the significant difference in the genotype and allele frequencies of g.IVS1-26G░>░C SNP in the intronic region of *TNP2* in patients with varicocele compared with controls and also because of the high conservation of this SNP position during evolution, this SNP may be involved in some important processes associated with the expression of this gene like mRNA splicing, but the exact mechanism is not clear.

## 1 Introduction

One of the main identifiable causes of male infertility is varicocele caused by veins dilatation of of the pampiniform plexus of the spermatic cord in the scrotum (1). Varicocele is a progressive lesion that usually occurs in the left testicle (2).

The prevalence of this disorder is about 15–20% among the general population (3). Despite all evidence, including alteration in testicular histology and normal function of Sertoli and Leydig cells and also changes in semen parameters that occur as a result of varicocele (4, 5), there is still a debate about the direct mechanism responsible for the impact of varicocele on spermatogenesis and male fertility potential (6–9).

One of the important factors in the process of fertilization and normal development of the embryo is sperm DNA integrity (10). Several studies have shown that infertile men compared with fertile men display higher levels of sperm DNA damages (11–15).

During spermiogenesis, the sperm nucleus will undergo a major rearrangement, in which the histones are replaced by positively charged protamines (PRMs). The replacement of histones by PRMs in sperm DNA is supported by other proteins such as transition nuclear proteins (TNPs) (16, 17). This event makes sperm DNA highly condensed and protects the sperm genome integrity that is important for normal sperm function and fertility (18, 19).

*TNP2* gene is located on human chromosome 16p13.3 (20, 21). The pathogenic mutations in the *PRM* and *TNP* genes could be one of the reasons for these altered levels (19). Several studies have demonstrated that in men with varicocele, the sperm DNA integrity is significantly reduced and also the amount of DNA damage is increased compared with healthy controls (12, 13, 22).

Therefore, because of the critical role of TNPs and PRMs in sperm DNA integrity, these proteins could be one of the best goals for study in varicocele condition. So in this study, we decided to analyze the *TNP2* gene mutations in Iranian infertile varicocele patients.

## 2 Material & Methods

### 2.1 Participants

This study was a case-control study. The study included 156 Iranian infertile males with varicocele and a history of infertility of ≥ 2 yr. The healthy donors included 150 fertile Iranian men who had a successful pregnancy, and normal spermogram.

The varicocele diagnosis and blood sample collection were performed by the urologist from 2011 to 2015. Semen analysis was performed according to the WHO criteria (23). Sperm parameters are listed in Table I that include sperm count, rapid (grade a), slow (grade b), and non-progressive (grade c) motility, and sperm morphology. The mean age of infertile men with varicocele and normal controls was 28.3░±░5.63 and 29.7░±░6.14 (Mean░±░SD), respectively, and were match to each other.

**Table I:** Mean and standard deviation of sperm parameters of patients and participants of the variants, SIFT and PolyPhen (sequence homology-based tools) were used.

Variables	Varicocele (n░=░156)	Control (n░=░150)	P-value^*^
Age	28.3░±░5.63	29.7░±░6.14	0.790
Count (mil.ml^-1^)	46.20░±░44.29	106.20░±░58.39	0.000
Rapid motility (%) or grade a	11.03░±░9.72	26.32░±░6.89	0.000
Slow motility (%) or grade b	32.01░±░8.14	29.12░±░3.68	0.615
Non-progressive motility (%) or Grade c	17.56░±░8.32	10.41░±░3.01	≤ 0.001
Normal morphology (%)	26.80░±░15.35	41.98░±░8.13	≤ 0.001

* Differences between variables with normal distribution were analyzed the Chi-square goodness-of-fit test

Peripheral Blood samples of patient and control groups were collected in EDTA-coated tubes in the Research and Clinical Center for Infertility, Shahid Sadoughi University of Medical Sciences, Yazd, Iran and were stored at -20░°C until analysis.

### 2.2 Semen analysis

Semen samples collection were performed by masturbation after 2–4 days of sexual abstinence. Semen analysis was completed according to the guidelines of the WHO criteria (World Health Organization, 1999) and Diff quick staining was applied for sperm morphology score (24, 25). Azoospermic samples were excluded from the study. Normal values were sperm motility ≥ 50% (a░+░b), sperm concentration ≥ 20  × 106 per ml, and normal sperm forms > 30%.

### 2.3 The assessment of sperm nuclear chromatin

Three techniques of cytochemical staining were used for sperm nuclear chromatin evaluation. For distinguishing the anomalies of sperm chromatin condensation, aniline blue was used according to Auger et al. Briefly, air-dried smears of fresh semen samples of a patient fixed in glutaraldehyde and phosphate buffer at room temperature were then stained with aqueous AB stain in acetic acid. Then stained and unstained spermatozoa were evaluated by light microscopy (Olampus, Tokyo, Japan).

The rate of sperm nuclear chromatin condensation was measured via toluidine blue (TB) staining (1). In this procedure, air-dried sperm smears were fixed in fresh ethanol-acetone and then hydrolyzed in HCl. Then the samples were rinsed in distilled H_2_O and finally stained with TB. The scores of chromatin quality of spermatozoa according to this procedure, included 0, good chromatin; 1, mild abnormal chromatin; 2, severe chromatin abnormality (1). So, spermatozoa with scores 1 and 2 were considered as TB░+░or abnormal chromatin, score 0 as a TB- or spermatozoa with normal chromatin.

In situ acid-induced denaturation of sperm nuclear DNA were measured by Acridine Orange (AO) staining. In this staining, air-dried sperm smears were fixed in Carnoy’s solution and then each sample was stained in AO. Samples were evaluated on the fluorescent microscope. The green (normal double-stranded DNA) and orange/red (abnormally denatured DNA) fluorescence spermatozoa percentage were calculated according to the recommended method provided by Talebi et al (15).

### 2.4 Mutation screening of *TNP2* gene

Genomic DNA was extracted from the blood samples with DNA extraction kit (CinnaClone, Tehran, Iran). Two PCR primer sets were designed and optimized by using the software Gene Runner version 3.05 (Hastings Software, Hastings, NY, USA) to proliferate the two exons of *TNP2* gene (Table II). PCR amplification was carried out at 94░°C for 5░min, followed by 35 cycles of denaturation at 94░°C for 30░sec, annealing at 61░°C (Exon 1) and 62░°C (Exon 2) for 40░sec, and extension at 72░°C for 40░sec, followed by a final extension for 5░min. PCR of each sample was set in a 0.5░ml tube using 100░ng of total DNA, 10░pM of each primer, 200░μM of dNTPs, 1X PCR buffer containing 2.5░mM MgCl2, and 1 U Taq DNA polymerase (CinnaClone, Tehran, Iran).

**Table II:** Primers used for amplification with PCR

Primer sequence (5'-3')	Primer size (bp)	Exon	Amplicon size (bp)
*F1*: AGC CTT CCT ATC ACC CAC AC	20	1	440
*R1*: GGT CTG CTC TCC ATC ATC TG	20		
*F2*: TAC AGG AGG TCA AGG AGG TCA C	22	2	127
*R2*: ACA CGC AGG AAC AAG CCA AG	20		

Single strand conformation polymorphism (SSCP) technique was used to analyse the unknown mutations because it's a simple and sensitive procedure (26). All PCR products from cases and controls were analyzed by SSCP. For the SSCP analysis, 5░μl of the PCR products were mixed with 10░μl of SSCP loading buffer. The samples were denatured at 95░°C for 5░min and then rapidly cooled on ice to prevent renaturation; 10░μl of this mixture was loaded onto the polyacrylamide gels. The PCR products of exon 1 with a size of 440 bp was loaded onto 8% (w/v) polyacrylamide gel for 22░hr at 100░V and 14░mA, and the PCR products relating to exon 2 with a size of 127 bp were loaded onto 10% (w/v) polyacrylamide gel for 15░hours at 110░V and 16░mA. The gels were stained using the standard silver staining procedure to reveal the bands of single-strand DNA, and then the gels were wrapped in cellophane for preservation. Direct sequencing of DNA from samples with altered band pattern in the SSCP was done by a commercial agency (Macrogene Seoul, South Korea) to identify mutations.

### 2.5 Bioinformatics analysis

The MEGA4 software was used for multiple sequence alignment to find the amount of homology of the sequences that have been obtained in the study and with all other sequences of the other species. As the structure of human transition protein 2 (TP2) has not yet been solved, the homology-based structural prediction service Protein Model Portal (http://www.proteinmodelportal.org/) (27) was used to generate the structure by submitting the human TP2 sequence (UniProt: Q05952). Using PyMol software, the effect of altered residue in the protein structure was evaluated. Also, to determine the pathogenicity of the variants, SIFT and PolyPhen (sequence homology-based tools) were used.

### 2.6 Ethical consideration

The ethics committee of the Biology Department of Yazd University approved all the plans and protocols of this study and each participant was fully informed of the objectives and content of it.

### 2.7 Statistical analysis

The *TNP2* gene SNPs were included in three genotype categories (wild-type, heterozygote, and homozygote SNPs) and then grouped into two categories with heterozygotes and homozygote variants combined because of the dominant, the recessive, and the over-dominant models of inheritance observed for these polymorphisms.

In order to clarify the relationship between the two study groups, we used the Chi-square goodness-of-fit test, and the values of p░<░0.05 were regarded as statistically significant. Also, a Chi-square test was performed for determining if the case and control groups demonstrate Hardy-Weinberg equilibrium. Odds ratio (OR) was calculated to measure the strength of the association observed. Multiple logistic regression analysis models were used to evaluate the haplotypes as possible risk factors. This statistical analysis was performed by SPSS (Statistical Package for the Social Sciences, version 20, SPSS Inc., Chicago, IL, USA).

## 3 Results

The results of aniline blue staining show that AB-reacted spermatozoa (AB+) rates in varicocele and control groups were significant (p░≤░0.001). With regard to the AO test, DNA damage in sperm nuclei from infertile men with varicocele was a significant difference control group (p░=░0.001). Also, the results of TB staining showed a significant difference between control and varicocele groups (p░≤░0.001).

**Figure 1:**
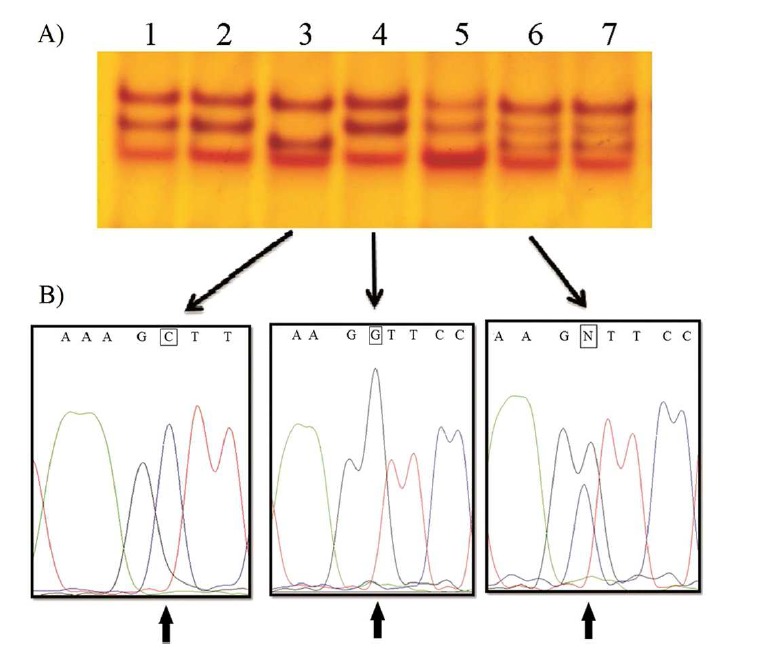
(A) The SSCP patterns of exon 2 of *TNP2* gene. (B) The results of direct sequencing of exon 2 of *TNP2* gene revealed g.IVS1-26G░>░C (rs8043625). Lanes 3, 4, and 6 show G/G, C/C, and C/G genotypes, respectively. Also, lanes 1, 2, and 5 have G/G genotype and lane 7 similar to lane 6 has C/G.

After the amplification of DNA fragments that isolated from blood samples of 156 patients and 150 controls using PCR and then SSCP procedures, some altered conformation patterns were detected by the SSCP analysis for the fragments of exons 1 & 2 of the *TNP2* gene. Finally, the samples of these altered conformation patterns were sequenced (Figure 1). The results of sequencing showed three variants at positions g.IVS1-26 G░>░C (rs8043625), c.301C░>░T (rs34904070), and c.391C░>░T (rs11640138) of this gene. c.301C░>░T mutation changes the Arginine to Cysteine at position 101 (p.R101C) and c.391C░>░T changes the Arginine to Tryptophan at position 131 (p.R131░W) of TP2 protein.

Genotype distribution in the control group for the all polymorphisms was in Hardy-Weinberg equilibrium. No significant differences were observed in genotype and allele frequencies of c.301C░>░T and c.391C░>░T variants. The distribution of genotype frequencies between Varicocele patients and controls wasn’t statistically significant, differences neither in the co-dominant nor in the dominant and recessive models (Tables III and IV). But for g.IVS1-26G░>░C SNP, the genotype frequencies of the G/G, G/C, and C/C were as 25.6%, 44.9%, and 29.5%, respectively, in cases and 50.7%, 37.3%, and 12%, respectively, in controls. The logistic regression analysis showed a significant association of the g.IVS1-26G░>░C SNP and Varicocele according to the dominant, recessive, and over-dominant models (dominant model OR: 2.98, 95% CI: 1.84–4.82; p░=░0.000, recessive model OR: 3.07, 95%CI: 1.68–5.59); p░=░0.000, and over-dominant model OR: 1.37, 95% CI: 0.86–2.16; p░≤░0.001) (Table V).

**Table III:** Genotype distribution and relative allele frequencies of rs34904070 (c.301C░>░T) polymorphism in the *TNP2* gene among Varicocele and control group

rs34904070 (c.301C░>░T)	Patients (n░=░156)	Controls (n░=░150)	OR (95% CI)	P-value
Codominant model				
CC	78 (50%)	84 (56%)	1 (ref.)	
CT	66 (42%)	60 (40%)	1.10 (0775–1.560)	0.593
TT	12 (7.7%)	6 (4%)	2.00 (0.571–5.329)	0.166
Dominant model				
CC	78 (50%)	84 (56%)	1 (ref.)	0.293
CT░+░TT	78 (50%)	66 (44%)	1.27 (0.81–2.00)	
Recessive model				
CC░+░CT	144 (92.3%)	144 (96%)	1 (ref.)	0.165
TT	12 (7.7%)	6 (4%)	2.00 (0.73–5.47)	
Over-dominant model				
CC░+░TT	90 (57.7%)	90 (60%)	1 (ref.)	0.681
CT	66 (42.3%)	60 (40%)	1.10 (0.70–1.73)	
Allele frequency				
C	228 (76%)	222 (71.15%)		
T	72 (24%)	90 (28.85%)	0.779 (0.543–1.117)	0.199

* P-value by Chi-square goodness-of-fit test

**Table IV:** Genotype distribution and relative allele frequencies of rs11640138 (c.391░T░>░C) polymorphism in the *TNP2* gene among Varicocele and control group

rs11640138 (c.391░T░>░C)	Patients (n░=░156)	Controls (n░=░150)	OR (95% CI)	P-value
Codominant model				
TT	54 (34.6%)	60 (40%)	1 (ref.)	
CT	70 (44.9%)	64 (42.7%)	1.094 (0.779–1.535)	0.604
CC	32 (20.5%)	26 (17.3%)	1.231 (0.734–2.065)	0.432
Dominant model				
TT	54 (34.6%)	60 (40%)	1 (ref.)	
CT░+░CC	102 (65.4%)	90 (60%)	1.26 (0.79–2.00)	0.330
Recessive model				
TT░+░CT	124 (79.5%)	124 (82.7%)	1 (ref.)	
CC	32 (20.5%)	26 (13.3%)	1.23 (0.69–2.19)	0.477
Over-dominant model				
TT░+░CC	86 (55.1%)	86 (57.3%)	1 (ref.)	
CT	70 (44.9%)	64 (42.7%)	1.09 (0.70–1.72)	0.697
Allele frequency				
T	184 (61.33%)	178 (57.05%)		
C	116 (38.67%)	134 (42.95%)	0.837 (0.606–1.157)	0.286

* P-value by Chi-square goodness-of-fit test

**Table V:** Genotype distribution and relative allele frequencies of rs8043625 (g.IVS1-26G░>░C) polymorphism in the *TNP2* gene among Varicocele and control group

rs8043625 (g.IVS1-26G░>░C)	Patients (n░=░156)	Controls (n░=░150)	OR (95% CI)	P-value
Codominant model				
GG	40 (25.6%)	76 (50.7%)	1 (ref.)	
GC	70 (44.9%)	56 (37.3%)	1.250 (0.880–1.776)	0.213
CC	46 (29.5%)	18 (12.0%)	2.556 (1.482–4.407)	0.001
Dominant model				
GG	40 (25.6%)	76 (50.7%)	1 (ref.)	
GC░+░CC	116 (74.4%)	74 (49.3%)	2.98 (1.84–4.82)	0.000
Recessive model				
GG░+░GC	110 (70.5%)	132 (88.0%)	1 (ref.)	
CC	46 (29.5%)	18 (12.0%)	3.07 (1.68–5.59)	0.000
Over-dominant model				
GG░+░CC	86 (55.1%)	94 (62.7%)	1 (ref.)	
GC	70 (44.9%)	56 (37.3%)	1.37 (0.86–2.16)	0.000
Allele frequency				
G	208 (69.30%)	150 (48.1%)	2.442 (1.754–3.400)	p < 0.001^*^
C	92 (30.70%)	162 (51.9%)		

P-value by Chi-square goodness-of-fit test

Table VI shows the haplotype frequencies of three SNPs in two groups. The results of multiple logistic regression analyses revealed that the haplotypes (C/C/T) and (C/T/T) were a risk factor in patients with varicocele compared to controls (OR░=░3.278, p░≤░0.001, and OR░=░9.304, p░=░0.038, respectively).

**Table VI:** Haplotype analysis of *TNP2* polymorphisms with Varicocele patients and in controls

Haplotype	Patients (n░=░156)	Controls (n░=░150)	OR (95% CI)	P-value
G/C/T*	79 (50.64)	105 (70.00)	1 (ref.)	
G/C/C	21 (13.46)	22 (14.67)	1.269 (0.652–2.468)	0.483
G/T/T	7 (4.49)	3 (2.00)	3.101 (0.777–12.372)	0.108
G/T/C	3 (1.92)	2 (1.33)	1.994 (0.325–12.217)	0.455
C/C/T	37 (23.72)	15 (10.00)	3.278 (1.682–6.389)	0.000
C/C/C	7 (4.47)	2 (1.33)	9.878E-9 (0.000)	0.998
C/T/T	1(0.64)	1 (0.67)	9.304 (1.122–77.165)	0.038
C/T/C	1 (0.64)	0 (0.00)	-	-

OR: Odds ratio; CI, Confidence interval

G/C/T haplotype were shown for g.IVS1-26G░>░C/c.301C░>░T/c.391░T░>░C polymorphisms, respectively

P-value by Chi-square goodness-of-fit test

## 4 Discussion

Recent studies showed a significant relationship between male infertility and reduced TP2 gene expressions (28, 29). During spermiogenesis, sperm nucleus are compacted, and for this reason, chromatin must be changed by the replacement of the common histones with PRMs (which are derived from histone H1) and TNPs (30, 31). Since nucleotide changes in the genes that code these nuclear proteins lead to male infertility in mice, it suggests that the replacement of the common histones with these nuclear proteins is the most important event during spermiogenesis (5). In the present study, to examine *TNP2* gene alterations in relation to human male infertility, we assessed the prevalence of *TNP* gene SNPs in infertile patients. Varicocele is one of the most common causes of seminal alteration (6). New studies support the hypothesis that the varicocele is a multifactorial disorder. There are many etiopathogenic factors that reveal this condition, but many etiologic and pathophysiologic aspects of this disorder need to be revealed (32). Our results of the evaluation of sperm nuclear chromatin showed that spermatozoa with abnormal DNA and immature chromatin in the varicocele patients were a higher proportion than those of the control group. Also, we found two reported missense mutations, including c.301C░>░T (rs34904070) and c.391C░>░T (rs11640138) in our patients. We report the non-significant difference in genotype and allele frequencies of these polymorphisms between an infertile man with varicocele and controls. The c.301C░>░T and c.391C░>░T mutations in *TNP2* gene previously have been described by Imken et al in infertile men by PCR and direct sequencing. Also, they did not find any significant association with the infertile phenotype (33).

**Figure 2:**
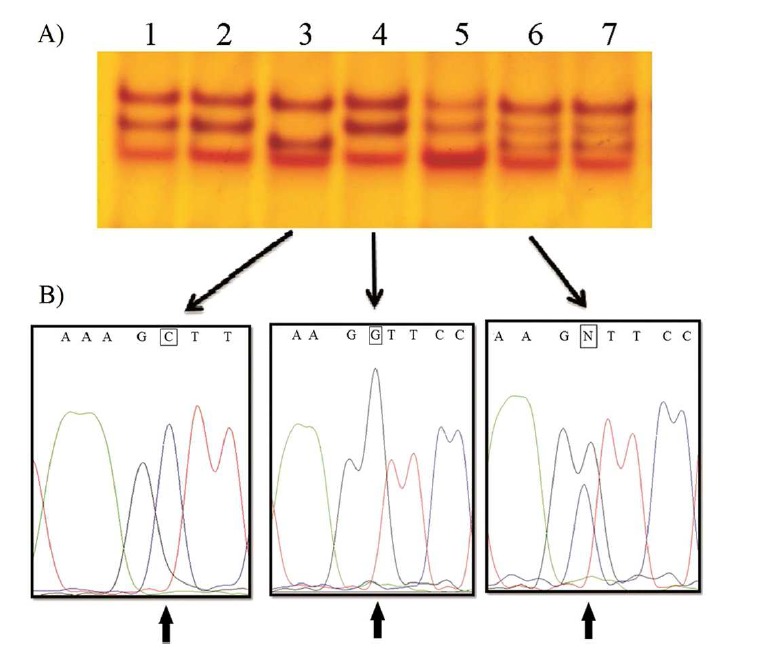
A three-dimensional structure model prediction of TP2 protein using the Protein Model Portal server and showing a close-up of the R101 (A) and C101 residues (B), R131 (C) and W131 (D) using PyMol.

Also, we identified the g.IVS1-26G░>░C (rs8043625) in the intronic region of this gene in both cases and controls that showed a significant difference in incidences between the patient and the control group. Siasi et al studied the relationship between polymorphisms of *TNP2* gene with male infertility in 96 idiopathic infertile men with azoospermia or oligospermia and 100 normal control men in Iranian population. Their results show that the g.IVS1-26G░>░C was not associated with male infertility (34). *Miyagawa et al* evaluated the mutations of *TNP1* and *TNP2* genes in male infertility in the Japanese population. In this study, more than 10 SNPs were found in these genes in which the prevalence g.IVS1-26G░>░C in infertile males was not significantly different from that in fertile controls (35). However, biochemical analyses have proven that TNP2 increases the melting temperature of DNA and leads to DNA compacts to nucleosomal cores, which shows that it is a DNA-condensing protein (36). These analyses represent that this protein plays an important role in chromatin remodeling during spermiogenesis and for this reason, a few amino acid changes are tolerated in TNP2. However, the importance of TNP2 in human sperm formation is not fully understood. In our study, a three-dimensional structure model prediction of TP2 protein using the Protein Model Portal server has been made. The prediction of the tertiary structure of TP2 protein through in-silico analysis indicated that p.R101 and p.R131 are parts of a β strand located in a conserved region among several mammalian species (Figure 2). Predicted results of the pathogenicity of these variants with SIFT and PolyPhen tools are also summarized in Table VII. IVS1-26G░>░C is a known single nucleotide polymorphism that was identified in the intronic region of this gene (35). *TNP2* sequence is poorly conserved during the evolution, and the levels of *TNP2* expression and its protein vary among species (35). But the g.IVS1-26G░>░C polymorphism shows that they are conserved among other species and we also found a significant difference in the genotype and allele frequencies in patients with varicocele compared to controls. In addition, the haplotype analysis suggests combining genetic variations are associated with increased risk in patients with varicocele compared to controls (Table VI).

**Table VII:** Predicted results of the pathogenicity of rs34904070 (P.101 R░>░C) and rs11640138 (P.131 R░>░W) variants with SIFT and PolyPhen tools

Predictor	Variation	Score	Prediction
*SIFT*	P.101 R░>░C	0.02	AFFECT PROTEIN FUNCTION
	P.131 R░>░W	0.00	AFFECT PROTEIN FUNCTION
*PolyPhen*	P.101 R░>░C	0.096	BENIGN
	P.131 R░>░W	0.886	POSSIBLY DAMAGING

## 5 Conclusion

These findings indicate that this SNP (rs8043625) in varicocele patients may affect in intron splicing and may cause human male infertility in varicocele patients, but the exact molecular effects of this polymorphism on varicocele condition are still controversial. Next researches using linkage studies of SNPs in pedigrees and a larger population of infertile men should confirm the causal link between *TNP2* gene polymorphisms and male infertility.

## Conflict of Interest

The authors declare that they have no conflict of interests.
